# PDK4 rescues high-glucose-induced senescent fibroblasts and promotes diabetic wound healing through enhancing glycolysis and regulating YAP and JNK pathway

**DOI:** 10.1038/s41420-023-01725-2

**Published:** 2023-11-25

**Authors:** Zhouji Ma, Youjun Ding, Xiaofeng Ding, Haining Mou, Ran Mo, Qian Tan

**Affiliations:** 1 Department of Burns and Plastic Surgery, Nanjing Drum Tower Hospital Clinical College of Nanjing Medical University, NO. 321, Zhongshan Road, Nanjing, Jiangsu China; 2 Department of Burns and Plastic Surgery, Nanjing Drum Tower Hospital Clinical College of Jiangsu University, NO. 321, Zhongshan Road, 210008 Nanjing, Jiangsu China; 3 Department of Emergency Surgery, The Fourth Affiliated Hospital of Jiangsu University (Zhenjiang Fourth People’s Hospital), Zhenjiang, China; 4 Department of Dermatologic Surgery, Shanghai Skin Disease Hospital, Tongji University School of Medicine, Shanghai, China; 5 Department of Burns and Plastic Surgery, Nanjing Drum Tower Hospital, the Affiliated Hospital of Nanjing University Medical School, NO. 321, Zhongshan Road, Nanjing, Jiangsu China; 6 Department of Burns and Plastic Surgery, Anqing Shihua Hospital, Nanjing Drum Tower Hospital Group, 246002 Anqing, China

**Keywords:** Diabetes complications, Senescence

## Abstract

During the process of wound healing, fibroblasts migrate to the wound site and perform essential functions in promoting cell proliferation, as well as synthesizing and secreting the extracellular matrix (ECM). However, in diabetic wounds, senescent fibroblasts exhibit impaired proliferative capacity and fail to synthesize essential ECM components. Pyruvate dehydrogenase kinase 4 (PDK4), a key enzyme regulating energy metabolism, has been implicated in modulating cellular senescence and fibroblast function. However, its specific role in diabetic wounds remains poorly understood. In this study, we conducted a series of in vivo and in vitro experiments using STZ-induced diabetic mice and human dermal fibroblasts. We evaluated cellular senescence markers, including SA-β-gal, P53, P16, P21, and PAI-1, as well as senescence-associated secretory phenotype (SASP) factors. Finally, we observed that PDK4 increased in normal wound healing, but its expression was insufficient in diabetic wounds. Significantly, the overexpression of PDK4 demonstrated the potential to accelerate diabetic wound healing and improve the senescence phenotype both in vivo and in vitro. Furthermore, our study elucidated the underlying mechanism by which PDK4 improved the senescent phenotype through the enhancement of glycolysis and regulation of YAP and JNK pathway. The effect was dependent on metabolic reprogramming and subsequent reduction of reactive oxygen species (ROS), which was mediated by PDK4. Overall, our findings highlight the potential of PDK4 as a promising therapeutic target for addressing diabetic wounds.

## Introduction

Diabetes is a chronic metabolic disease that poses a significant threat to both the physical and mental well-being of individuals. As of 2019, it was estimated that there were 463 million people worldwide living with diabetes [1]. Approximately 20% of diabetic patients will eventually develop diabetic wounds, primarily in the form of diabetic foot ulcers (DFU) [2]. Elderly diabetic patients, in particular, bear a heavy burden of DFUs [3]. Regrettably, there has been limited progress in reducing the amputation rate associated with DFUs [4]. Therefore, finding new approaches to treat diabetic wounds is an urgent matter.

During wound healing, fibroblasts migrate to the wound bed to proliferate and contribute to the synthesis and secretion of the extracellular matrix (ECM). Additionally, fibroblasts express cytokines, growth factors, and undergo differentiation into myofibroblasts, facilitating wound contraction and promoting wound closure [5]. However, these vital functions are impaired within diabetic microenvironments [6]. The underlying mechanisms for this impairment include cellular senescence induced by hyperglycemia, oxidative stress, mitochondrial dysfunction, and DNA damage [7].

Pyruvate dehydrogenase kinases (PDKs) are critical enzymes located in the mitochondria that negatively regulate the activity of the pyruvate dehydrogenase (PDH) complex by phosphorylating its subunit. This regulation plays a crucial role in glucose utilization and lipid metabolism [8]. Among mammals, there exist four isoenzymes of PDKs (PDK 1–4) with varying binding affinities, phosphorylation site specificities, and tissue distributions [9]. PDK4 is the predominant isoform found in tissues with high energy demands, including skin wound tissues during the repair process. Recent studies have demonstrated that PDK4 is also involved in regulating cellular senescence phenotypes. Reduced PDK4 expression has been observed in aging cardiomyocytes, which is associated with metabolic maladaptation [10, 11]. Besides, putrescine delays postovulatory aging of mouse oocytes by upregulating PDK4 expression and improving mitochondrial activity [12]. MuRF1 deficiency improves age-related dysfunction of lipid metabolism by upregulating PDK4 localization into mitochondrial [13]. In fibroblast functions, PDK4 has also been implicated. PDK4 deficiency leads to decreased proliferation and increased apoptosis of canine dermal fibroblasts under starvation conditions [14]. Additionally, PDK2, another isoenzyme, promotes the proliferation of periorbital fibroblasts by stimulating glycolysis [15]. Previous research has reported downregulated expression of PDK4 in dermal fibroblasts of diabetic patients [16]. However, our understanding of the relationship between PDK4 and dermal fibroblasts, as well as its role in senescence, remains limited. Furthermore, the involvement of PDK4 in the senescent phenotype during diabetic wound healing remains unknown.

In this study, we observed that PDK4 increased in normal wound healing, but its expression was insufficient in diabetic wounds. PDK4 expression decreased in a high-glucose environment. Overexpression of PDK4 reversed high-glucose induced senescence in human dermal fibroblasts (HDFs) and accelerated wound healing in diabetic mice. Mechanistically, we confirmed that PDK4 improved the senescent phenotype via enhancement of glycolysis and regulation of Yes-associated protein (YAP) and c-Jun N-terminal kinase (JNK) pathway, which was dependent on metabolic reprogramming and subsequent reduction of reactive oxygen species (ROS) mediated by PDK4. Overall, the identification of PDK4-mediated improvement of the senescent phenotype sheds new light on the treatment of non-healing diabetic wounds.

## Results

### Diabetic wounds display a prolonged healing process and senescent phenotype

**Fig. 1:**
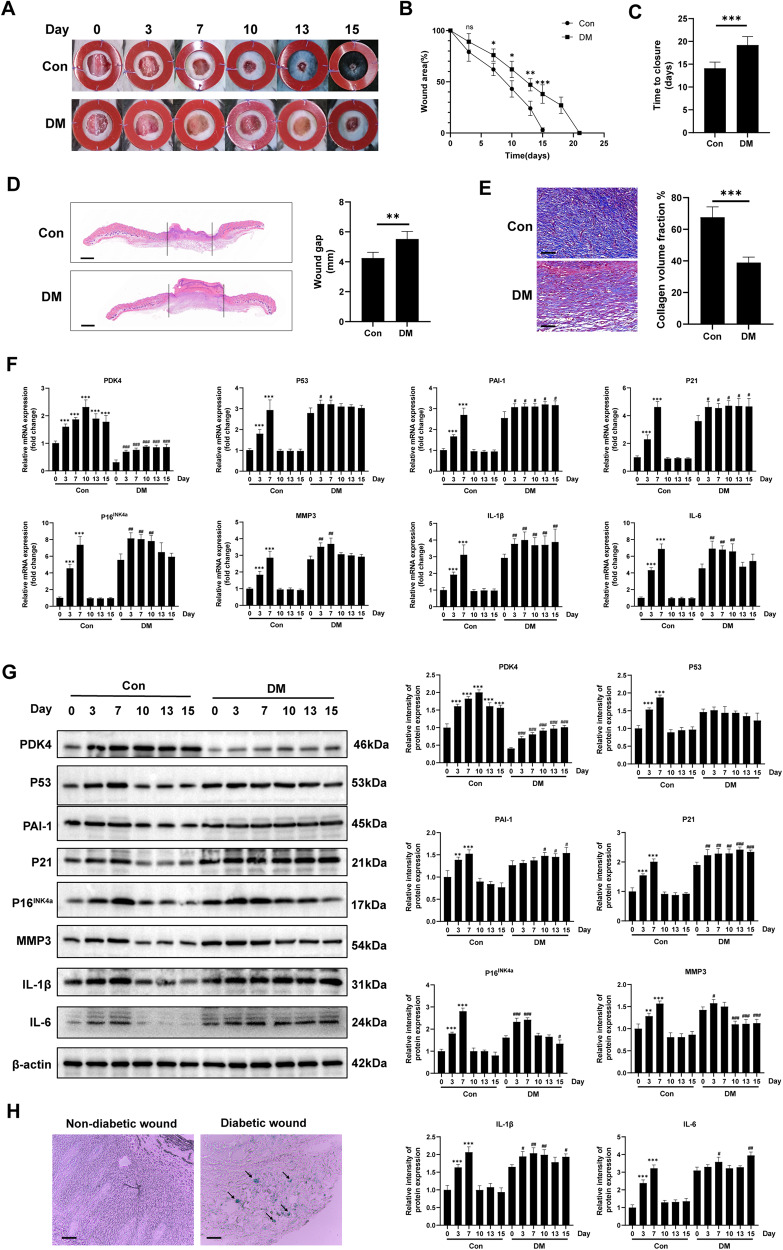
Diabetic wounds display a prolonged healing process and senescent phenotype. **A** Gross examination of the wound area in diabetic mice and the control group at the indicated time points. **B** The skin wound area of diabetic mice and the control group during the wound healing process (*n* = 4, expressed as the percentage of the initial wound area). **C** Wound healing time of two groups (*n* = 4). **D** HE staining (scale bar, 1 mm) and **E** Masson’s trichrome staining (scale bar, 100μm) of day-10 skin wounds of two groups. **F**, **G** PDK4, senescence markers and SASP-related markers were detected by RT-qPCR and Western blot in skin wound tissues of diabetic mice and the control group (*n* = 4; *, **, ***, compared to Day 0 in the control group; #, ##, ###, compared to Day 0 in the DM group). **H** Representative images of SA-β-Gal^+^ (blue) cells, depicted by black arrows from skin wound tissues of a diabetic patient (59 years old, a 3-mm skin biopsy was taken from the 10-day wound edge on the lower extremity) and a non-diabetic patient (51 years old, a 3-mm skin biopsy was taken from the 10-day wound edge on the lower extremity caused by a traffic accident) (scale bar, 50 μm). The data are shown as the mean ± SD. *P < 0.05, **P < 0.01, ***P < 0.001.

To compare the difference in wound healing between diabetic wounds and normal wounds, we created 10-mm full-thickness wounds on the dorsal skin of diabetic/non-diabetic mice. As shown in Fig. 1A, B, the wounds of diabetic mice exhibited a slower healing process compared to those of the control group. Importantly, the wound areas of diabetic mice were significantly larger than those of the control group on day 7 (*P* < 0.05), 10 (*P* < 0.05), 13 (*P* < 0.01), and 15 (*P* < 0.001). Even after 15 days of healing, the wounds in diabetic mice remained visibly apparent, whereas the wounds in the control group were fully healed. Consequently, the diabetic mice exhibited prolonged wound closure time (Fig. 1C). Furthermore, hematoxylin and eosin (HE) staining of the wounds revealed a wider gap in the diabetic mice (Fig. 1D). Masson trichrome staining (Fig. 1E) demonstrated a remarkable decrease in collagen deposition in the skin wound tissues of diabetic mice, with disordered arrangement of collagen fibers. To explore the role of PDK4 in wound healing, we detected its expression pattern during wound healing process both in control group and DM group. We observed that PDK4 increased during normal wound healing process and reached its peak on day 10 after wounding. However, in diabetic wounds, although pdk4 increased slightly after wounding, its expression was still insufficient (Fig.1F, G). Additionally, we observed that the senescence markers (P53, P21, and P16^INK4a^) increased rapidly during the early normal wound healing and reached the peak on the day 7, but decreased rapidly to the baseline level after day 10 (Fig.1F, G). Nevertheless, they showed a sustained high level of expression in diabetic wounds. Notably, SERPINE1 (PAI-1), a well-known marker of fibroblast senescence [17], and senescence-associated secretory phenotype (SASP) factors, including MMP-3, IL-6, and IL-1β, represented a similar expression pattern (Fig. 1F, G). PDK4 reached its peak on the 10th day of wound healing, but the senescence markers quickly returned to the baseline level. At this time, the wound healing is during the proliferation and remodeling stage, when fibroblasts play an important role. Thus, we chose this time point to study their regulating relationship and detect the senescence phenotype and functions of fibroblasts in the subsequent study. More importantly, increased senescence-related β galactosidases (SA-β-GAL) was also confirmed in human diabetic wounds (Fig. 1G). Taken together, these findings indicate the persistent accumulation of senescent cells in impaired diabetic wound healing.

### PDK4 expression decreases in skin wound tissues and dermal fibroblasts of diabetic patients and mice

**Fig. 2:**
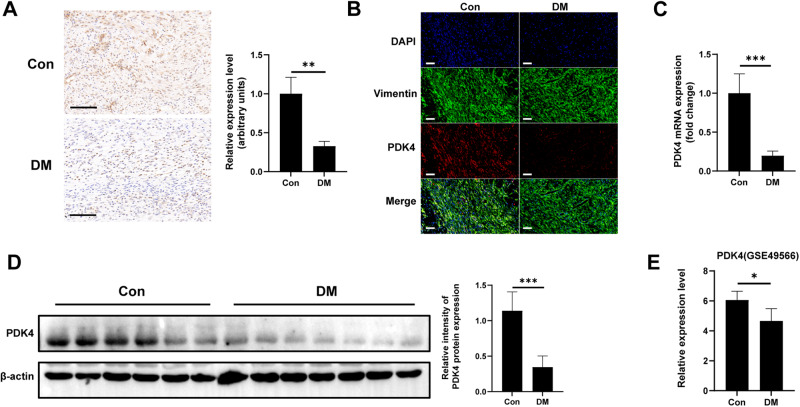
PDK4 expression decreases in skin wound tissues and dermal fibroblasts of diabetic mice and patients. **A** Immunohistochemical analysis of PDK4 in skin wound tissues of diabetic mice or control group on day-10 after wound (*n* = 4). **B** Immunofluorescence double staining of PDK4-expressing (red) Vimentin+ cells (green) in skin wound tissues of diabetic mice or control group (scale bar, 20 μm). **C** PDK4 level detected by RT-qPCR in human skin wound tissues (*n* = 12, 14 respectively) **D** Representative Western blot images analyzing PDK4 in in human skin wound tissues. **E** PDK4 expression levels reported in GSE49566 (high-throughput transcriptomics data) in dermal fibroblasts of normal or diabetic patients. Data are shown as the mean ± SD. *P < 0.05, **P < 0.01, ***P < 0.001, ns not significant.

To further verify the low expression of PDK4 in diabetic wounds, we employed immunohistochemical staining (Fig. 2A) and immunofluorescence double staining (Fig. 2B). The results revealed a reduction in PDK4 expression in skin tissues and specifically in fibroblasts during the process of skin wound healing in diabetic mice. To further validate these observations, we collected skin wound tissues from twelve non-diabetic individuals and fourteen diabetic patients with DFU for the analysis of PDK4 expression. Consistently, the results of RT-qPCR (Fig. 2C) and Western blot analysis (Fig. 2D) in human samples aligned with the findings in the mouse samples. Additionally, by analyzing the dataset GSE49566 [16], we discovered that PDK4 expression was lower in dermal fibroblasts of diabetic patients (Fig. 2E). Collectively, these data provided robust evidence indicating the downregulation of PDK4 expression in the wound tissues and dermal fibroblasts of both diabetic patients and mice.

### PDK4 overexpression accelerates diabetic wound healing, improves the senescence phenotype, and induces metabolic reprogramming in skin wound tissues

**Fig. 3:**
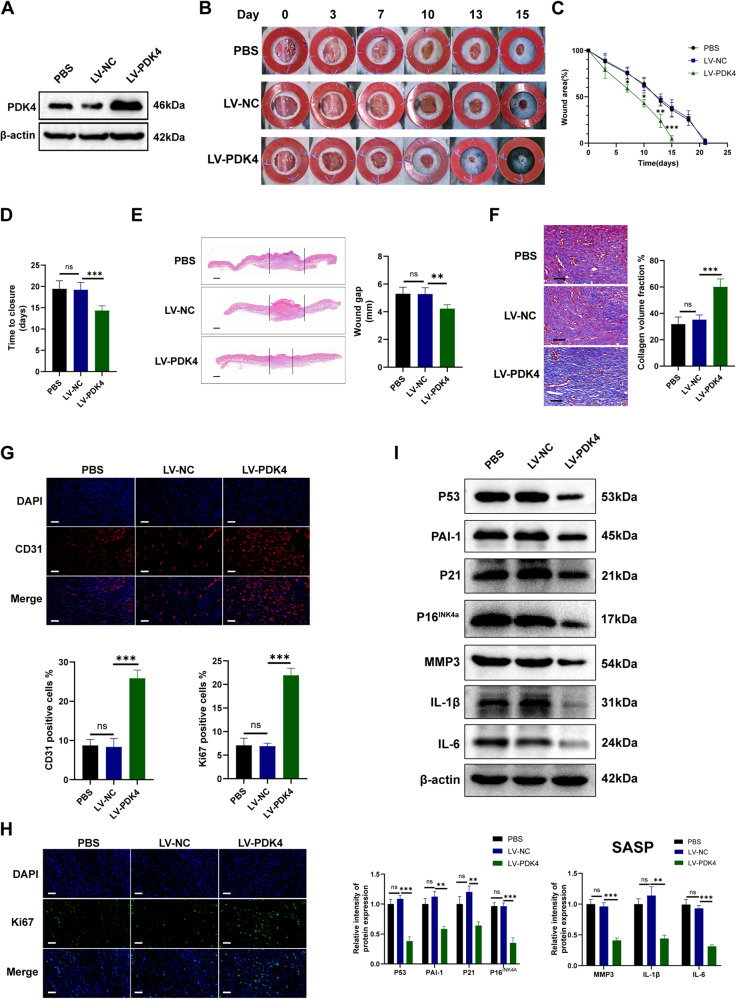
PDK4 overexpression accelerates diabetic wound healing and improves the senescence phenotype. **A** Western blot analysis of PDK4 to confirm efficient transduction. **B** Gross examination of the wound area at the indicated time points. **C** The skin wound area during the wound healing process (*n* = 4, expressed as the percentage of the initial wound area) **D** Wound healing time of two groups (*n* = 4). **E** HE staining (scale bar, 1 mm) and **F** Masson’s trichrome staining (scale bar, 100 μm) of day-10 skin wounds. **G** Angiogenesis evaluated by CD31 immunohistochemical analysis (*n* = 4, scale bar, 50 μm). **H** Proliferation evaluated by Ki67 immunohistochemical analysis (*n* = 4, scale bar, 50 μm). **I** Senescence markers and SASP-related protein detected by western blot in mice skin wound tissues (*n* = 4). Data are shown as mean ± SD of three independent experiments, *P < 0.05, **P < 0.01, ***P < 0.001, ns not significant.

**Fig. 4:**
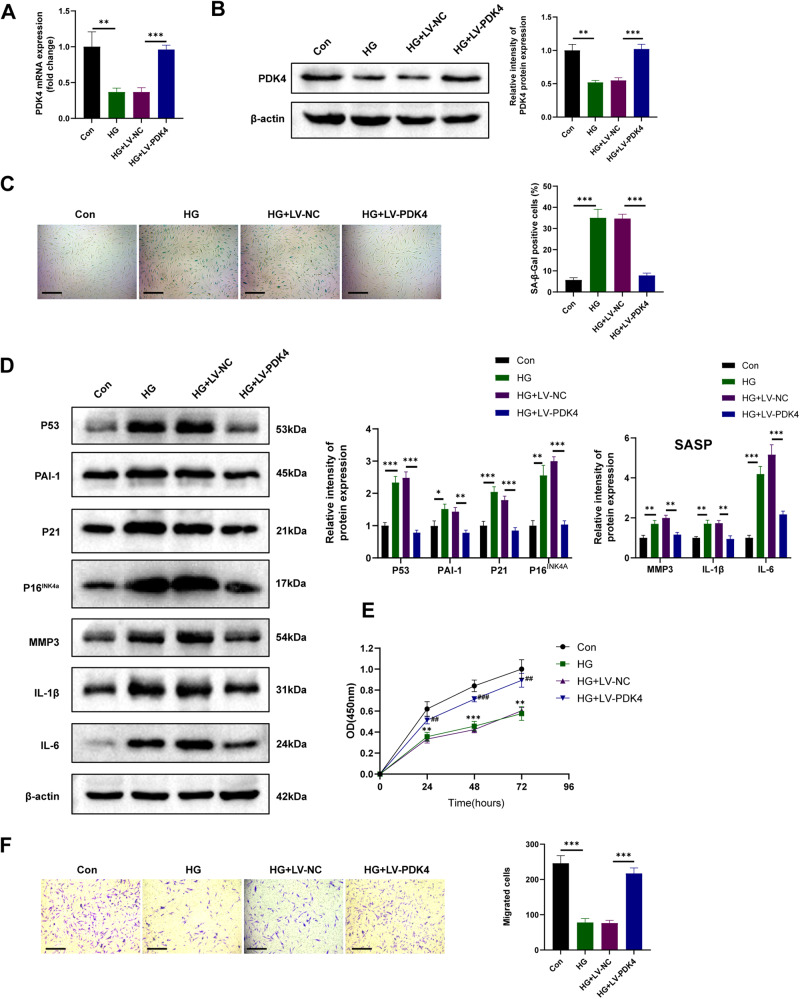
PDK4 overexpression inhibits HDFs high-glucose-induced senescence. **A**, **B** RT-qPCR and western blot analysis of PDK4 in different groups of HDFs (*n* = 3). **C** SA-β-GAL staining images of different groups of HDFs (*n* = 3). **D** Senescence markers and SASP-related protein detected by western blot in HDFs (*n* = 3). **E** Cell proliferation assessed by cell counting kit-8 assay (*n* = 3, **P < 0.01, ***P < 0.001, compared to the Con group; ##*p* < 0.01, ###*p* < 0.001, compared to the HG + LV-NC group;). **F** Transwell assay images and quantitative analysis of the migration of HDFs (*n* = 3, scale bar, 200 μm). The data are shown as the mean ± SD. *P < 0.05, **P < 0.01, ***P < 0.001.

Subsequently, we assessed the effect of lentiviral-transduced PDK4 (LV-PDK4) overexpression on diabetic wound healing. Firstly, we confirmed efficient transduction with Western blot (Fig. 3A), which revealed a significant increase in PDK4 expression compared with the LV-NC and PBS groups. PDK4 overexpression facilitated the process wound healing in diabetic mice, as evident from the subsequent macroscopic analysis of wound photos (Fig. 3B). The diabetic mice treated with LV-PDK4 exhibited a smaller residual wound area (Fig. 3C) at the indicated time points and achieved a shorter wound-healing time (Fig. 3D). Concurrently, the LV-PDK4 group demonstrated a narrower gap of wound (Fig. 3E) and improved deposition of collagens (Fig. 4F). Furthermore, LV-PDK4 treatment resulted in increased cell proliferation rates, as assessed by ki67 staining (Fig. 3G) and enhanced angiogenesis, as assessed by CD31 immunofluorescent staining (Fig. 3H). Most notably, PDK4 overexpression led to a decrease in the expression of senescence markers, including P53, P21, P16^INK4a^ and fibroblast-specific marker PAI-1 in the skin wound tissues of diabetic mice (Fig. 3I). Additionally, the LV-PDK4 group exhibited reduced levels of SASP-related proteins, including MMP-3, IL-6, and IL-1β (Fig. 3I).

PDK4 is a key molecule in regulating glucose metabolism, therefore we next focused on the metabolic shift in the skin wound tissues of diabetic mice. Accordingly, we examined the phosphorylation level of PDH, a target protein of PDK4. Immunofluorescence analysis showed that phosphorylation of PDH was significantly increased in the PDK4-overexpressed mice (Fig. S1A). Meanwhile, the reduction of PDH enzyme activity was observed (Fig. S1B). PDK4 profoundly affected mitochondrial respiration and reduced oxygen consumption rate (OCR) in diabetic wounds (Fig. S1C). Additionally, PDK4 overexpression also resulted in increased lactate production (Fig. S1D), indicating increased glycolysis. All these data showed the metabolic reprogramming induced by PDK4 in diabetic wounds.

### PDK4 overexpression inhibits HDFs high-glucose-induced senescence via enhancing glycolysis

PDK4 expression was found to be downregulated in HDFs exposed to a high-glucose environment. However, the introduction of LV-PDK4 reversed this downregulation, as depicted in Fig. 4A, B. To evaluate the impact of high glucose on cell senescence, we conducted SA-β-gal assays. Remarkably, overexpression of PDK4 significantly suppressed senescence induced by high glucose (Fig. 4C). In vitro experiments further revealed that PDK4 overexpression led to a reduction in the expression of senescence markers, namely P53, P21, P16^INK4a^, and the fibroblast-specific marker PAI-1, which were upregulated in response to high glucose (Fig.4D). Additionally, the LV-PDK4 group exhibited diminished levels of SASP-related proteins, including MMP-3, IL-6, and IL-1β (Fig. 4D), consistently aligning with the outcomes of in vivo experiments. Meanwhile, senescent HDFs exposed to high glucose demonstrated impaired proliferation and migration capacities. However, CCK8 (Fig. 4E) and Transwell (Fig. 4F) experiments demonstrated that PDK4 overexpression improved the proliferation and migration abilities of HDFs under high glucose conditions.

Furthermore, metabolic reprogramming was also observed in PDK4-overexpressed HDFs. Similarly, we noticed an increased phosphorylation level of PDH (Fig. S2A) and decreased PDH enzyme activity in vitro (Fig. S2B). PDK4 overexpression induced declined OCR (Fig. S2C) and elevated extracellular acidification rate (ECAR; Fig. S2D), accompanied by more lactate production (Fig. S2E) in high-glucose induced senescent HDFs. It was reported that the existence of a glycolytic phenotype antagonizes entry into senescence [18]. Then, we further investigated the relationship between senescence and PDK4-induced enhanced glycolysis. We treated PDK4-overexpressed HDFs with 2-DG (15 mM, 1 h), a glycolytic inhibitor, and PDK4-induced anti-senescence effect in HDFs was abrogated according to the results of SA-β-gal assays (Fig. S2F) and senescence markers protein level (Fig. S2G). The improved proliferation (Fig. S2H) and migration (Fig. S2I) abilities of HDFs induced by PDK4 overexpression were also attenuated. All these data suggested that the anti-senescence effect of PDK4 on HDFs was mediated by an increase in glycolysis at least partially.

### PDK4 suppresses high-glucose-induced senescence in HDFs through activation of YAP

**Fig. 5:**
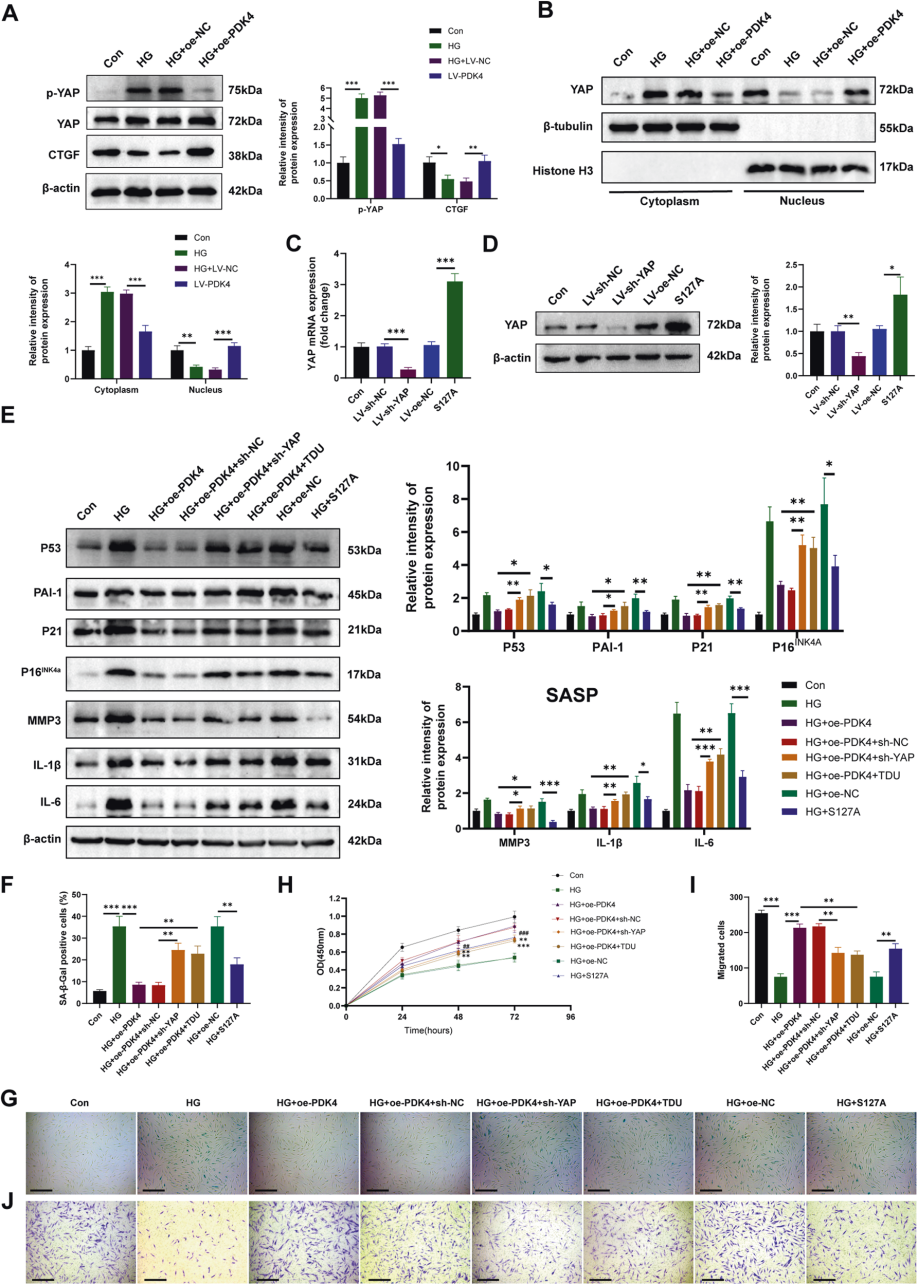
PDK4 suppresses high-glucose-induced senescence in HDFs through YAP nuclear translocation. **A** Western blot analysis of protein level of p-YAP, YAP, and CTGF in HDFs (*n* = 3). **B** Level of YAP detected by western blot in cytoplasmic and nuclear extraction. Histone H3 was used as a nuclear internal reference, and β-tubulin was used as a plasma reference (*n* = 3). **C**, **D** RT-qPCR and western blot analysis of YAP to verify the efficiency of knockdown and overexpression (*n* = 3). **E** Senescence markers and SASP-related protein detected by western blot in HDFs (*n* = 3). **F**, **G** SA-β-GAL staining images of different groups of HDFs (*n* = 3). **H** Cell proliferation assessed by cell counting kit-8 assay (*n* = 3, **P < 0.01, ***P < 0.001, compared to the HG+oe-PDK4 group; ##*p* < 0.01, ###*p* < 0.001, compared to the HG+oe-NC group;). **I**, **J** Transwell assay images and quantitative analysis of the migration of HDFs (*n* = 3, scale bar, 200μm). Data are shown as mean ± SD. *P < 0.05, **P < 0.01, ***P < 0.001; compared to the ED-EVs group, ###*p* < 0.001.

Yes-Associated Protein (YAP) plays an important role in skin tissues and dermal fibroblasts senescence [19]. Our findings revealed that PDK4 overexpression in HDFs resulted in reduced YAP phosphorylation and increased nuclear accumulation of YAP under high-glucose conditions (Fig. 5A, B). This was accompanied by the activation of connective tissue growth factor (CTGF) (Fig. 5A), a downstream effector protein of YAP known to promote fibroblast proliferation and collagen deposition [20]. To ascertain the critical role of YAP as a mediator of PDK4-induced anti-senescence effect on HDFs, we employed lentiviral shRNA to knock down YAP levels in fibroblasts and confirmed the efficiency of the shRNA-mediated inhibition (Fig. 5C, D). Subsequently, we observed a partial attenuation of the PDK4-induced anti-senescence effect, as evidenced by the expression levels of senescence markers and SASP-related proteins (Fig. 5E), and confirmed through SA-β-gal assay (Fig. 5F, G). Additionally, the pro-proliferation and migration effects of PDK4 on HDFs were also nullified (Fig. 5H–J). Furthermore, we employed the YAP inhibitor, super TDU (TDU), to validate the role of YAP, which disrupts the interaction between YAP and TEAD [21]. Notably, the inhibition of YAP partially reversed the effects of PDK4 on HDFs treated with 50 nM TDU for 24 h (Fig. 5E–J). Additionally, we conducted overexpression of S127A, a constitutively-active mutant of YAP, in fibroblasts. Following transfection, we assessed the senescence markers, proliferation, and migration of HDFs. Encouragingly, we observed that the anti-senescence and promotional effects of PDK4 on fibroblast proliferation and migration were partly recapitulated by S127A overexpression (Fig. 5E–J). Collectively, these results strongly suggest that the PDK4-induced anti-senescence effect in HDFs can be partially attributed to the activation of YAP.

### PDK4 inhibits high-glucose-induced senescence in HDFs by dephosphorylating JNK

**Fig. 6:**
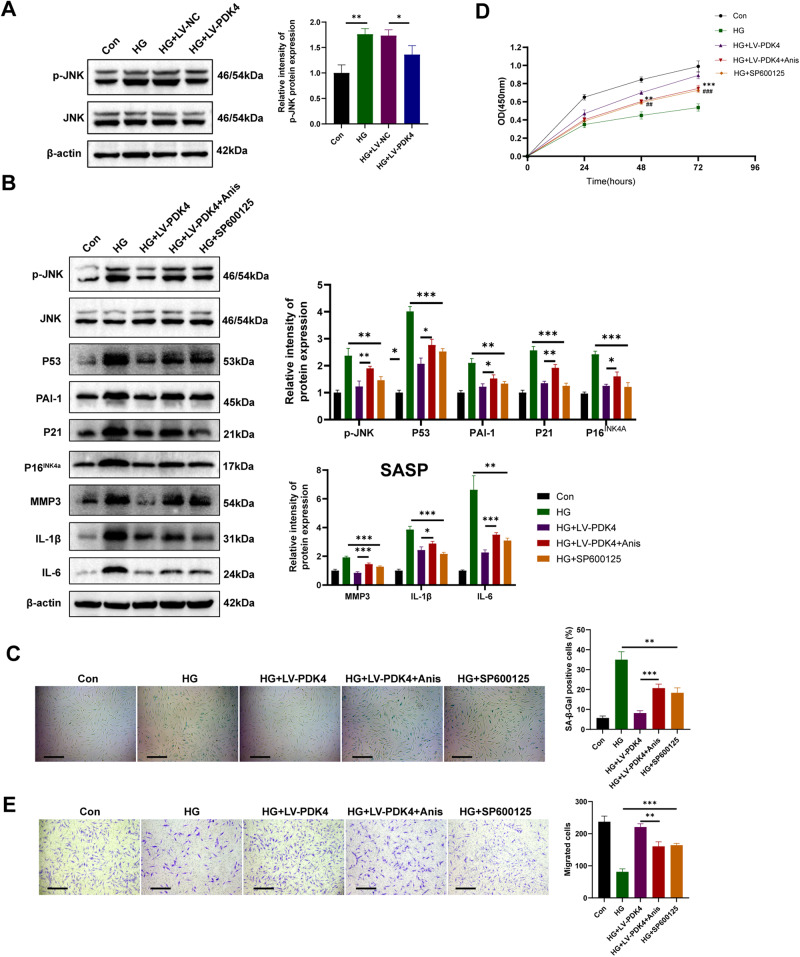
PDK4 inhibits high-glucose-induced senescence in HDFs by dephosphorylating JNK. **A** Western blot analysis of protein level of p-JNK, JNK in HDFs (*n* = 3). **B** Senescence markers and SASP-related protein detected by Western blot in HDFs (*n* = 3). **C** SA-β-GAL staining images of different groups of HDFs (*n* = 3). **D** Cell proliferation assessed by cell counting kit-8 assay (*n* = 3, **P < 0.01, ***P < 0.001, compared to the HG + LV-PDK4 group; ##*p* < 0.01, ###*p* < 0.001, compared to the HG group;). **E** Transwell assay images and quantitative analysis of the migration of HDFs (*n* = 3, scale bar, 200 μm). Data are shown as mean ± SD. *P < 0.05, **P < 0.01, ***P < 0.001.

The involvement of the c-Jun N-terminal kinase (JNK) pathway in the aging process of skin and fibroblasts has been previously documented [22]. Initially, we observed an upregulation in the phosphorylation levels of JNK under high-glucose conditions. However, overexpression of PDK4 resulted in the reversal of JNK phosphorylation (Fig. 6A). To elucidate the role of JNK, we employed Anisomycin (Anis) as a JNK activator and SP600125 as a JNK inhibitor. Strikingly, treatment with Anisomycin (10uM, 24 h) partially attenuated the anti-senescence effects exerted by PDK4, as demonstrated by the expression levels of senescence markers and SASP-related proteins (Fig. 6B), which was further confirmed by SA-β-gal assay (Fig. 6C). Furthermore, the pro-proliferative and migratory effects of PDK4 on HDFs were also abrogated (Fig. 6D, E). Conversely, treatment with the JNK inhibitor SP600125 (10uM, 24 h) partially mimicked the anti-senescence effects induced by PDK4 in HDFs (Fig. 6B–E). All these data suggest that JNK pathway is involved in PDK4-induced anti-senescence effects on HDFs.

### PDK4-induced ROS reduction mediates activation of YAP and JNK dephosphorylation

**Fig. 7:**
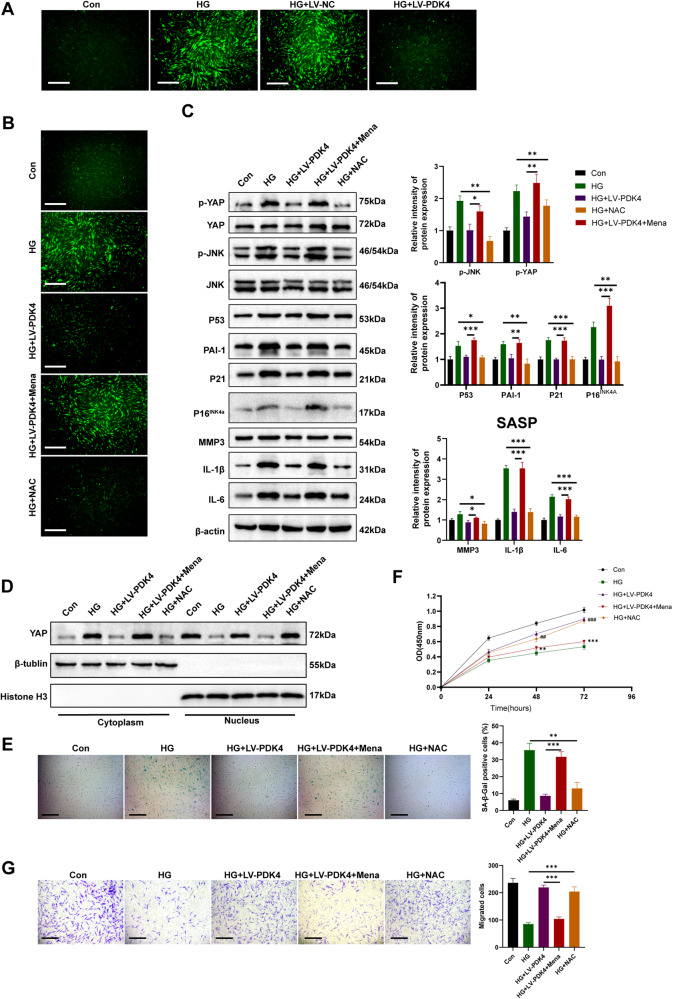
PDK4-induced ROS reduction mediates YAP nuclear translocation and JNK dephosphorylation. **A**, **B** Representative fluorescence images of reactive oxygen species (ROS) staining in HDFs (scale bar, 200 μm). **C** p-YAP, YAP, p-JNK, JNK, senescence markers and SASP-related protein detected by western blot in HDFs (*n* = 3). **D** Level of YAP detected by Western Blot in cytoplasmic and nuclear extraction. Histone H3 was used as a nuclear internal reference, and β-tubulin was used as a plasma reference (*n* = 3). **E** SA-β-GAL staining images of different groups of HDFs (*n* = 3). **F** Cell proliferation assessed by cell counting kit-8 assay (*n* = 3, **P < 0.01, ***P < 0.001, compared to the HG + LV-PDK4 group; ##*p* < 0.01, ###*p* < 0.001, compared to the HG group;). **G** Transwell assay images and quantitative analysis of the migration of HDFs (*n* = 3, scale bar, 200 μm). Data are shown as mean ± SD, *P < 0.05, **P < 0.01, ***P < 0.001, ns not significant.

Gao et al. recently demonstrated the inhibitory effect of PDK4 overexpression on pyruvate dehydrogenase (PDH) activity through PDH phosphorylation following subarachnoid hemorrhage, resulting in reduced production of reactive oxygen species (ROS) [23]. Furthermore, it has been reported that ROS acts as an upstream signal in regulating the YAP [24] and JNK [25] signaling pathways, which are involved in senescence regulation. Therefore, in this study, we aimed to investigate whether the anti-senescence effects of PDK4 in human dermal fibroblasts (HDFs) were mediated through its impact on oxidative stress. To determine the role of PDK4 in oxidative stress regulation, we first examined ROS production in HDFs under high glucose conditions (Fig. 7A). Our findings revealed that PDK4-reduced ROS production induced by high glucose. To investigate the involvement of ROS in PDK4-mediated anti-senescence signaling, we utilized the ROS generator Menadione (Mena) and the ROS scavenger NAC to modulate intracellular ROS levels (Fig. 7B). Upon restoration of PDK4-reduced ROS levels by Menadione (25 μM, 2 h), we observed an increase in cytoplasmic localization of YAP and JNK phosphorylation (Fig. 7C, D). Furthermore, Menadione treatment restored the senescence phenotype that was inhibited by PDK4 (Fig. 7C, E), and attenuated the proliferation and migration capacity of HDFs (Fig. 7F, G). Conversely, treatment with NAC (1 mM 24 h) resulted in YAP nuclear translocation and JNK dephosphorylation (Fig. 7C), mimicking the anti-senescence effects of PDK4 on HDFs (Fig. 7E–G). Collectively, these results indicated that PDK4-induced ROS reduction is responsible for YAP nuclear translocation and JNK dephosphorylation.

### PDK4 overexpression promotes activation of YAP and dephosphorylation of JNK in diabetic mice

**Fig. 8:**
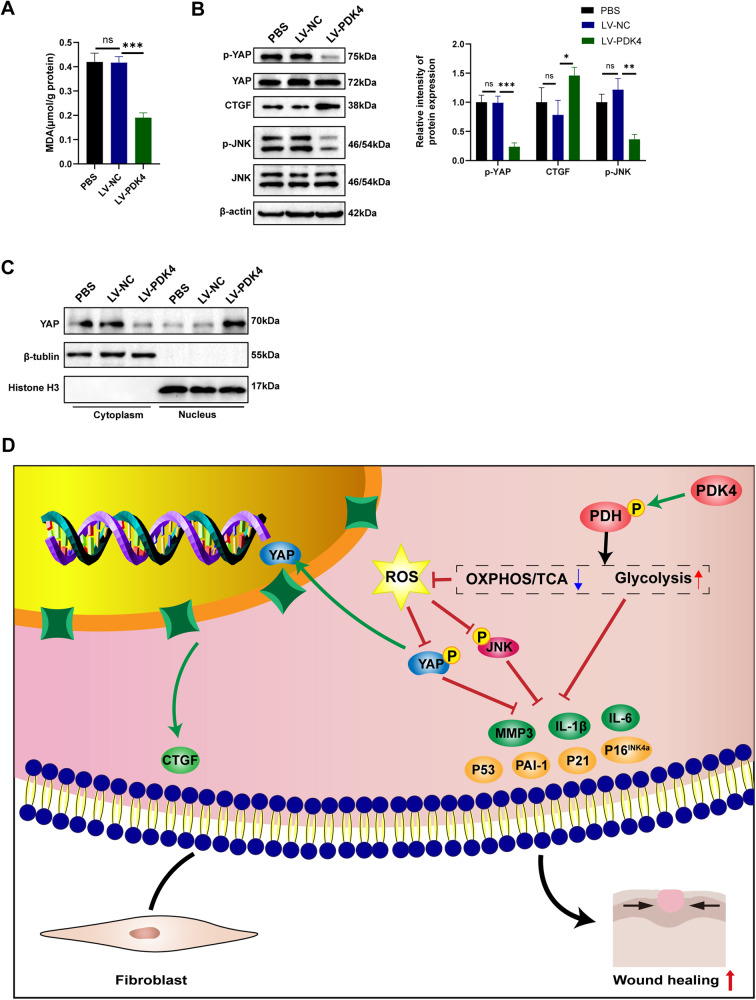
PDK4 overexpression promotes YAP nuclear translocation and dephosphorylation of JNK in diabetic mice. **A** Quantitative analysis of the MDA in skin wound tissues (*n* = 4). **B** p-YAP, YAP, CTGF, p-JNK, and JNK detected by western blot in skin wound tissues (*n* = 4). **C** Level of YAP detected by western blot in cytoplasmic and nuclear extraction. Histone H3 was used as a nuclear internal reference, and β-tubulin was used as a plasma reference (*n* = 4). **D** Concise model of PDK4 in inhibiting HDFs senescence and accelerating wound healing. Data are shown as mean ± SD, *P < 0.05, **P < 0.01, ***P < 0.001, ns not significant.

To further elucidate the regulatory mechanisms underlying the accelerated wound healing induced by PDK4, we examined samples from skin tissues from diabetic mice. We evaluated the oxidative stress levels in the wound tissue using the MDA test and observed a decrease in oxidative stress upon PDK4 overexpression (Fig. 8A). Moreover, PDK4 overexpression resulted in a reduction in the phosphorylation level of YAP and facilitated its nuclear translocation, leading to the activation of its downstream target CTGF (Fig. 8B, C). Notably, the phosphorylation level of JNK was also decreased in response to PDK4 overexpression (Fig. 8B).

## Discussion

Chronic wound in individuals with diabetes pose a significant clinical challenge, and their prevalence is expected to rise due to the increasing incidence of diabetes [26]. In the early stage of wound healing, transient senescence phenotype induction is essential for normal wound healing [27]. Notably, the persistent presence of local cellular senescence constitutes a significant characteristic associated with impaired wound healing in diabetic patients [28]. Typically, wound healing progresses through distinct phases of inflammation, proliferation, and tissue remodeling, with fibroblasts playing a pivotal role in the latter two stages. However, senescent fibroblasts exhibit significant changes in gene expression patterns and functional behavior. They lose their proliferative capacity and fail to synthesize essential components of the ECM, which are critical for effective wound healing. Additionally, the release of factors by senescent fibroblasts promotes chronic inflammation and disrupts the function of neighboring cells [29]. In our study, we observed that senescent fibroblasts cultured under high-glucose conditions showed reduced expression of PDK4. Intriguingly, the overexpression of PDK4 in HDFs improved the senescent phenotype. We elucidated that this improvement was mediated by metabolic shift and regulation of YAP and JNK pathway (Fig. 8D). Our findings confirm that PDK4 can effectively ameliorate the senescence phenotype in fibroblasts induced by high glucose. Notably, these observations are consistent with previous reports suggesting a role for PDK4 in the regulation of cellular senescence [10, 11, 13, 14]. Moreover, overexpression of PDK4 accelerated wound healing in diabetic mice, decreased the expression of senescence markers in wound tissue, and promoted collagen deposition. These findings underscore the potential therapeutic value of targeting PDK4 in diabetic wound healing. In this study, we observed PDK4 expression decreased in diabetic skin and fibroblasts, but there is literature that states that the level of PDK4 is elevated in patients with type 2 diabetes and in animals and humans on a HFD [30]. Previous studied mainly focused on the expression of PDK4 in skeletal muscle, here, we paid attention to its role in skin tissues and wound healing. These opposite results may reflect the differences between the skin tissues and skeletal muscle tissues, skeletal muscle tissues demand a greater amount of energy to support their activities compared to skin tissues. Different tissues may have different metabolic adaptation pathways that regulate the expression of PDK4. The metabolic switches responsible for such expression differences across different tissues are not yet well elucidated, and further research is warranted.

Recent studies have shown that non-healing diabetic wounds may also be related to the metabolic mode. Decreased glycolysis and increased oxidative phosphorylation levels were observed in diabetic mice models of delayed wound healing [31]. Basu found that the expression of glycolysis-related enzymes was significantly downregulated in the wounds of unhealed diabetic mice [32]. Meanwhile, through metabolic reprogramming, MiR-210 changes the mode of cell metabolism from oxidative phosphorylation to glycolysis to enhance the migration ability of fibroblasts, thus promoting wound healing in diabetic mice [31]. Aging dermal fibroblasts undergo metabolic reprogramming, characterized by enhanced FA oxidation and reduced glycolysis [18]. However, a previous study suggested that the glycolysis level of senescent dermal fibroblasts is increased [33]. James et al. made an intriguing discovery regarding enhanced glycolysis in senescent human fibroblasts induced by γ-ray exposure. Their findings, as reported, reveal an unexpected facet of senescence, wherein glycolysis seemingly opposes the entry into senescence while concomitantly indicating a state of reduced oxidative stress, consistent with established senescent cell characteristics [33]. This is not strange, elevated glycolysis can be seen as a protective metabolic adaptation that cells employ in response to senescence. However, our own investigation reveals a distinct observation in high-glucose-induced senescent fibroblasts, wherein glycolytic activity is notably diminished. This peculiar trend is echoed in senescent endothelial cells as well [34]. Discrepancies such as these may stem from variances in senescence induction methods, the duration of induction, and the inherently dynamic nature of metabolic transitions. In our model of HDFs, it becomes evident that these cells have lost their ability to augment glycolysis as a defense against senescence, suggesting the possibility of divergent stages of senescence progression. Significantly, the key metabolic enzyme, PDK4, exhibited diminished expression levels in high-glucose-induced fibroblasts, mirroring the observed decline in glycolytic activity. Notably, the overexpression of PDK4 effectively augmented glycolysis, subsequently serving as a potent inhibitory factor against senescence induced by high glucose levels. These findings underscore the multifaceted nature of metabolic adaptations during senescence, offering new insights into the potential avenues for mitigating the deleterious effects of cellular senescence. Of course, further comprehensive investigations are warranted to elucidate the intricate nuances surrounding the characterization and categorization of senescence processes, particularly the intricate interplay between distinct senescence modalities and the resultant glycolytic phenotypes. Nevertheless, what is clear is that the existence of a glycolytic phenotype antagonizes entry into senescence [18, 33]. Our research yielded similar results. Enhanced glycolysis induced by PDK4 inhibited senescence in HDFs.

YAP, a versatile adaptor protein possessing a transcription coactivation domain, serves as a nuclear effector of the Hippo signaling pathway, which plays a crucial role in regulating organ size. In recent years, the involvement of YAP in the modulation of cellular senescence has garnered significant attention and has been extensively studied. YAP has been shown to prevent premature senescence of astrocytes and cognitive decline in Alzheimer’s disease by regulating CDK6 signaling [35]. In non-alcoholic fatty liver disease, curcumol inhibits ferritinophagy to restrain hepatocyte senescence through the YAP/NCOA4 axis [36]. Significantly, YAP also participates in the regulation of senescence in dermal fibroblasts [37]. Additionally, YAP knockdown has been associated with impaired skin wound healing [38]. In our study, we observed an increase in YAP phosphorylation levels and a decrease in nuclear localization in high glucose-induced senescent fibroblasts, which were effectively reversed by PDK4 overexpression. Furthermore, inhibition or knockdown of YAP partially abrogated the anti-senescence effect of PDK4, while overexpression of YAP partially mimicked the anti-senescence effect of PDK4. These findings underscore the critical mediating role of YAP in the process of PDK4-mediated regulation of senescence. Moreover, we discovered that PDK4-induced YAP nuclear translocation activated CTGF, a key factor known to promote fibroblast proliferation and collagen deposition.

JNK (c-Jun N-terminal kinase), a member of the mitogen-activated protein kinase (MAPK) family, also plays a significant role in cellular senescence. Elevated phosphorylation of JNK has been observed in aged skeletal muscle, and reducing JNK phosphorylation has been shown to mitigate sarcopenia in aged mice [39]. Similarly, increased JNK phosphorylation has been observed in senescent adipocytes, and reducing JNK phosphorylation has been found to improve the aging phenotype [40]. Notably, in Ultraviolet-induced senescent dermal fibroblasts, the phosphorylation level of JNK was found to be elevated, and treatment with the JNK inhibitor SP600125 resulted in a reduction in the expression level of SASP-related proteins [22, 41]. In our study, we observed increased JNK phosphorylation levels in high glucose-induced senescent fibroblasts, which were effectively reversed by PDK4 overexpression. Furthermore, inhibition of JNK partially abolished the anti-senescence effect of PDK4, while activation of JNK partially mimicked the anti-senescence effect of PDK4. These findings suggest that JNK plays a regulatory role in senescent fibroblasts under high glucose conditions and mediates the effects of PDK4.

ROS are highly reactive chemical species that contain oxygen, including peroxides, superoxide, hydroxyl radicals, singlet oxygen, and alpha-oxygen [42]. Cellular stress responses, such as ROS production and accumulation, can have hormetic effects [43, 44]. Specific increases in ROS levels have been shown to play a potentially crucial role in the induction and maintenance of cellular senescence processes [45]. Mitochondria are recognized as the primary intracellular sites for ROS production [46]. PDK4, in our study, phosphorylated PDH and reduced its activity, thereby inhibiting mitochondrial respiration and subsequently decreasing ROS generation. Additionally, ROS can function as signaling molecules in intracellular pathways. They have been identified as upstream molecules in the Hippo/YAP [47] and JNK [48] signaling pathway. Our findings align with previous studies indicating that the regulation of ROS can modulate the senescence phenotype of fibroblasts [45]. Furthermore, altered ROS levels also impact downstream signaling of YAP and JNK, indicating that ROS acts as a signaling molecule connecting PDK4 with the YAP/JNK pathways.

This study has certain limitations that warrant consideration. Firstly, diabetic wounds are characterized by a complex detrimental microenvironment that involves hyperglycemia, hypoxia, impaired vascularization, and chronic inflammation [49]. In this study, we focused on the behavior of fibroblasts in a “high-glucose” microenvironment. However, to fully replicate the complexity of the diabetic microenvironment, further experiments incorporating factors such as hypoxia, inflammatory mediators, and conditioned medium from actual wounds of patients with diabetes mellitus are required. Secondly, the underlying mechanism behind the low expression of PDK4 in high-glucose-induced senescent fibroblasts deserves further investigation. Elucidating the factors contributing to the downregulation of PDK4 in senescence will provide valuable insights into the regulatory mechanisms of cellular senescence in diabetic wounds. Addressing these questions in subsequent experiments will enhance our understanding of the intricacies of diabetic wound healing and the role of PDK4 in this process.

In conclusion, our findings shed light on the prolonged healing process and persistent senescent phenotype observed in diabetic wounds. We demonstrated that PDK4 expression is diminished in both diabetic wound tissues and fibroblasts. Notably, the overexpression of PDK4 exhibited the potential to expedite diabetic wound healing and ameliorate the senescence phenotype both in vivo and in vitro. Moreover, our study elucidated the underlying mechanism whereby PDK4 improved the senescent phenotype through enhancing glycolysis and regulating YAP and JNK pathway. Collectively, our findings suggest that PDK4 holds promise as a prospective therapeutic target for addressing diabetic wounds.

## Materials and methods

### Antibodies and reagents

Antibodies P53 (10442-1-AP), PAI-1 (13801-1-AP), PDK4 (12949-1-AP), p-PDH (29582-1-AP), PDH (18068-1-AP), P21 (10355-1-AP), P16^INK4a^ (10883-1-AP), MMP3 (17873-1-AP), β-actin (20536-1-AP), β-tubulin (10094-1-AP), Vimentin (10366-1-AP), IL-1β (16806-1-AP), IL-6 (21865-1-AP), CTGF (25474-1-AP), JNK (66210-1-Ig), and p-JNK (80024-1-RR) were purchased from Proteintech. Antibodies p-YAP (ab76252) and YAP (ab52771) were purchased from Abcam. Streptozotocin (STZ, S17049) was supplied by Yuanye Biotechnology Company. 2-Deoxy-d-glucose (2-DG, S4701), Super TDU (TDU, S8554), Anisomycin (Anis, S7409), SP600125 (S1460) and NAC (S1623) were procured from Selleckchem. Menadione (M2519) was purchased from Sigma-Aldrich. Isoflurane for animal experiments was purchased from RWD.

### Human skin samples

The human skin wound samples were collected from patients at the Affiliated Drum Tower Hospital of Nanjing University Medical School. Twelve samples were obtained from normal patients without diabetes, and fourteen were from patients with diabetes and DFU. A 3-mm section of full-thickness skin was taken from the wound edges of diabetic patients. The collection of human specimens and research protocols were approved by the Medical Research Ethics Committee of the Affiliated Drum Tower Hospital of Nanjing University Medical School (Ethics Approval Number: 2020-365-02) in compliance with the Declaration of Helsinki Principles. Informed consent was provided by all patients participating in the study. The clinical information of patients was shown in Table S1.

### Cell culture

Human dermal fibroblasts (HDFs) were collected from the abdominal skin of an abdominoplasty patient. HDFs were maintained at 37 °C, 5% CO2 in DMEM medium containing 5.5/35 mM D-glucose, 10% FBS, and 1% penicillin-streptomycin solution (Pen/Step) (Keygene Biotech, China). For high glucose (HG) stimulation experiments, cells were incubated with glucose at the final concentration 35 mM for 7 days [50]. The high glucose medium was prepared using D-glucose (Sigma-Aldrich, USA).

### Animal experiments

Male C57BL/6 mice, aged 6-8 weeks, were procured from Jiangsu Jicui Yaokang Biotechnology Co., Ltd. Animal experiments were permitted by the Medical School for Animal Use and Care Committee of Nanjing Drum Tower (Ethics Approval Number: 2020AE01093) and were performed according to the guidelines of NIH (USA).

To establish the diabetic mouse models, the mice were intraperitoneally injected with streptozotocin (STZ) dissolved in 0.1 M sodium citrate buffer. The injection was administered daily at a dose of 50 mg/kg (based on mouse body weight) for five consecutive days. Four weeks after the final STZ injection, fasting blood glucose (FGL) levels were measured. Mice with FGL values ≥16.7 mmol/L (300 mg/dL) were considered successfully induced diabetic mouse models. This study is not applicable for blinding.

Considering animal welfare and the number of animals traditionally used in prior studies within this field [27, 51], we have adopted *N* = 4 as the number of animals per data point.

To compare the wound healing differences between diabetic wounds and normal wounds, a full-thickness wound with a diameter of 10 mm was created using a sterile skin punch biopsy on the dorsal skin of the diabetic mice and the control group. To explore the expression pattern of PDK4 and senescence markers during wound healing process, mice were sacrificed at indicated time points and skin wound tissues were harvested for subsequent RT-qPCR and WB analysis.

To analyze the effect of PDK4 on diabetic wound healing, Lentiviral PDK4 overexpression (LV-PDK4) plasmids were intradermally injected into the dorsal skin of the diabetic mice. The diabetic mice treated with lentiviral negative control (LV-NC) plasmids and PBS were used as controls. After allowing 4 days for transgene expression, the mice were anesthetized and underwent surgery using a 10-mm sterile skin punch biopsy. Mice were randomly assigned to one of three groups using a drawing lots method.

The mice were positioned in a prone position and underwent surgery after appropriate anesthesia using isoflurane (induction 4%, maintenance 2%). A silicone splint (inner/outer diameter = 13/21 mm) was used to prevent skin contraction. The wound-healing process was documented through photographs. On day 10, a 3-mm section of full-thickness skin wound tissues was taken from the wound edges for subsequent analysis, including hematoxylin and eosin (HE) staining, Masson’s trichrome (MT) staining, reverse transcription-quantitative polymerase chain reaction (RT-qPCR), Western blot, immunohistochemical (IHC) analysis, and immunofluorescence (IF).

### RNA extraction and RT-qPCR

Total RNA was extracted using Beyozol reagent (Beyotime, Shanghai, China). RT -qPCR was performed using the Hieff qPCR SYBR Green Master Mix (Yeason, Shanghai, China) according to the manufacturer’s instructions. The mRNA levels of the target gene were quantified using the 2^−ΔΔCT^ method. The primer sequences for each target gene are provided in Table S2.

### Western blot

Total protein was extracted from human samples, HDFs, and mouse skin wound tissues using the Total Protein Extraction kit (Solarbio, Beijing, China). The protein concentration was determined using the BCA protein assay (Keygene Biotech, Nanjing, China). After separation on a 10% SDS-PAGE gel, the protein was transferred to PVDF membranes (Millipore, USA). The membranes were blocked with non-fat powdered milk (Beyotime, Shanghai, China) in TBST buffer and then incubated with primary and secondary antibodies. Finally, the protein blots were detected using ECL reagents (Vazyme, Nanjing, China) and recorded with the Tanon Chemi-Image System.

### Histological, immunofluorescent staining, and immunohistochemical analysis

The mice were sacrificed ten days after wounding, and the skin wound tissues were fixed with 4% paraformaldehyde. The tissues were either embedded in paraffin or frozen in liquid nitrogen for mRNA and protein analysis. For paraffin-embedded tissue, 5 mm thick sections were used for hematoxylin and eosin (HE) and Masson’s trichrome staining.

For immunofluorescent staining analysis, the paraffin-embedded skin sections were deparaffinized, hydrated, and antigen restored. Subsequently, they were washed with PBS and blocked with 2% BSA for 1 h. The sections were then incubated with the primary antibody overnight at 4 °C, washed three times for 5 min each with 0.1% Tween-20 in PBS (PBST), and stained with the secondary antibody for 1 h at room temperature in the dark. After rinsing, the slides were treated with 1 mg/mL DAPI (Keygene Biotech, Nanjing, China) in PBS for 5 min at room temperature to visualize the nuclei. Images were captured using a Leica Thunder microscope, and ImageJ software was used to analyze the expression levels of p-PDH, CD31, Ki67, PDK4, or Vimentin in different groups relative to DAPI.

For immunohistochemistry, sections were deparaffinized, hydrated, and endogenous peroxidase activity was quenched with 3% H2O2 for 10 min prior to antigen restoration. After washing three times with PBS, the sections were blocked with 2% BSA for 1 h and then incubated with the primary PDK4 antibody overnight at 4 °C. The sections were subsequently incubated with horseradish peroxidase (HRP)-conjugated secondary antibody for 1 h at room temperature and stained with DAB substrate solution. Images were captured using an Olympus FluoView FV3000 confocal microscope (Tokyo, Japan).

### Cell counting kit-8 assay

HDFs were harvested, resuspended, and seeded at a density of 1 × 10^3 cells/well in a 96-well plate. Ten µl of cell counting kit-8 solution (Vazyme, Nanjing, China) was added to each well at the indicated time. After a two-hour incubation, the absorbance (A) at 450 nm was measured using a microplate reader. A standard curve was generated to calculate the cell proliferation rate following the manufacturer’s instructions.

### Transwell assays

To assess HDFs’ migration capacity, Transwell chambers with 8 mm pore-size polycarbonate filters (Corning, USA) were used. HDFs were cultured in the upper chamber in DMEM without FBS, while the lower chamber contained DMEM with 10% FBS. After 24 h of incubation, migrated cells were fixed and stained with a 0.1% crystal violet solution for 30 min.

### Lentiviral transduction

HDFs were transduced with lentivirus carrying overexpression plasmids of PDK4, S127A, or short hairpin RNA against YAP (sh-YAP), along with their corresponding controls (oe-NC and sh-NC), following the protocol provided by the supplier. Briefly, HDFs were incubated in retroviral supernatant containing 5 μg/mL polybrene for 24 h. Forty-eight hours after infection, cells were selected with 2.5 μg/mL puromycin (Sigma-Aldrich) in the culture medium. Lentiviral plasmids (multiplicity of infection = 80) were obtained from GenePharma (Shanghai, China).

### Senescence associated-β-galactosidase expression assay

The expression of SA-β-Gal in HDFs and human skin tissues was observed using the SA-β-Gal staining kit (Beyotime, C0602) following the manufacturer’s instructions. The culture medium in the 6-well plate was discarded, and the cells were washed three times with 1× PBS. Fixative Solution (1 mL per well) was used to fix the cells for 15 min at room temperature. A Staining Solution Mix was prepared by combining Staining Solution A (10 μL), Staining Solution B (10 μL), Staining Solution C (930 μL), and X-Gal (50 μL) for each well. The cells were rinsed twice with 1× PBS and then treated with the Solution Mix (1 mL per well). The plate was covered and sealed with a sealing film to prevent CO2 from entering and incubated overnight at 37 °C. For human skin tissue samples, frozen sections were prepared and stained following similar protocols. The cells or tissues were examined under a microscope to detect the appearance of a blue hue indicative of SA-β-Gal expression.

### ROS generation evaluation

After culturing under the designated conditions, HDFs were washed with phosphate-buffered saline (PBS) and incubated with 10 μM 2′,7′-dichlorodihydrofluorescein diacetate (DCFH-DA, Beyotime, China) in a cell incubator (37 °C, 5% CO2) for 20 min. The accumulation of ROS in cells was visualized and imaged using a fluorescence microscope (Leica, Germany).

### MDA content detection

MDA content was detected using the Lipid Peroxidation MDA Assay Kit (Beyotime, S0131) according to the manufacturer’s instructions. Total skin protein was first extracted using the required reagents. Next, 100 μL of sample or standard sample was mixed with 200 μL of MDA detection solution in a tube. After heating at 100 °C for 15 min and centrifugation at 12,000×*g* at room temperature for 15 min, 200 μL of the resulting supernatant was transferred to a 96-well plate. Finally, the absorbance was measured at 530–540 nm at room temperature, and the results were calculated based on the standard curve.

### Measurement of lactate and PDH enzyme activity

Lactate concentrations in HDFs or skin wound tissues were quantified utilizing a lactate assay kit (Solarbio, Beijing, China), following the manufacturer’s provided protocol.

The activity of PDH in HDFs or skin wound samples was assessed employing a PDH enzyme activity assay kit (Solarbio, Beijing, China), following the manufacturer’s prescribed guidelines.

### Measurement of oxygen consumption rate and extracellular acidification rate

The assessment of basal OCR and ECAR was conducted using a Seahorse XF Analyzer (Agilent Technologies) in accordance with the XF Cell Mito Stress Test Kit and the XF Glycolytic Rate Test Kit. Sensor cartridges, designed for the measurement of oxygen flux, were pre-conditioned within an XF calibrator for a duration of 16–24 h, prior to commencing the experiments. The experiments were carried out in a 0% CO2 environment, maintained at 37 °C.

Skin wound tissues obtained on day 10 from mouse wounds were meticulously dissected and rinsed with an unbuffered Krebs-Henseleit buffer (KHB) medium. Subsequently, these tissues were analyzed in an XF24 Islet Capture Microplate (Agilent Technologies). In each well, 450 μl of KHB medium was added and allowed to equilibrate for 30 min within a 0% CO2 incubator. The sensor cartridges were then affixed to the testing platform and run in the XF Analyzer, following an optimized OCR measurement protocol. Human dermal fibroblasts (HDFs) from each experimental condition were subsequently seeded into an XF24 Cell Culture Microplate (Agilent Technologies). Results were standardized based on protein concentration.

### Statistics analysis

Statistical analysis was performed using SPSS and GraphPad Prism software. All data are presented as mean ± standard deviation. Student’s t-test or one-way analysis of variance (ANOVA) was used to compare statistical differences between two or more groups. The significance level was set at *P* < 0.05.

### Supplementary information

Supplementary Information

Figure S1

Figure S2

## Data Availability

The datasets used in this study are available from the corresponding authors upon reasonable request.
