# Supplementary material for: PDK4 rescues high-glucose-induced senescent fibroblasts and promotes diabetic wound healing through enhancing glycolysis and regulating YAP and JNK pathway
Source: Cell Death Discov. 2023 Nov 25;9:424. doi: 10.1038/s41420-023-01725-2 (PMC10674012; doi:10.1038/s41420-023-01725-2)
Supplement: Supplementary file 1 — Supplementary Information [file 41420_2023_1725_MOESM1_ESM.docx]

**Fig. S1** **PDK4 induces metabolic reprogramming in diabetic wounds**

**A** Immunofluorescence staining showing phosphorylation level of PDH in skin wound tissues of different groups (n=4, scale bar, 50μm). **B** PDH enzyme activity was determined in the skin wound tissues (n=4). **C** Oxygen consumption rate (OCR) of skin wound tissues was measured by Seahorse XF Analyzer (n=4). **D** Lactate contents were evaluated in the wound tissues (n=4).

**Fig. S2** **Glycolysis enhanced by PDK4 suppresses high-glucose-induced senescence in HDFs**

**A** Western blot analysis of protein level of p-PDH, PDH in HDFs (n=3). **B** PDH enzyme activity was determined in HDFs (n=3). **C-D** OCR and extracellular acidification rate (ECAR) were measured by Seahorse XF Analyzer (n=3). **E** Lactate contents were evaluated in HDFs (n=3). **F** SA-β-GAL staining images of different groups of HDFs (n=3). **G** Senescence markers and SASP-related protein detected by Western blot in HDFs (n=3). **H** Cell proliferation assessed by CCK-8 assay (n=3, ***P* < 0.01, ****P* < 0.001, compared to the HG+LV-PDK4 group; ##*p* < 0.01, ###*p* < 0.001, compared to the group;). **I** Transwell assay images and quantitative analysis of the migration of HDFs (n=3, scale bar, 200μm). The data are shown as the mean±SD. **P* < 0.05, ***P* < 0.01, ****P* < 0.001.

| **Table S1. Information of patients with diabetes and controls.** | | | | | | | | | |
| --- | --- | --- | --- | --- | --- | --- | --- | --- | --- |
| **Donor** | **Group** | **Gender** | **Age** | **HbA1c (%)** | **Donor** | **Group** | **Gender** | **Age** | **HbA1c (%)** |
| 1 | DFU | M | 66 | 10 | 1 | NW | M | 47 | - |
| 2 | DFU | F | 57 | 9.8 | 2 | NW | F | 52 | - |
| 3 | DFU | M | 72 | 7.9 | 3 | NW | F | 69 | - |
| 4 | DFU | M | 76 | 8.9 | 4 | NW | M | 59 | - |
| 5 | DFU | M | 49 | 9.4 | 5 | NW | F | 74 | - |
| 6 | DFU | F | 57 | 12.1 | 6 | NW | M | 56 | - |
| 7 | DFU | M | 65 | 9.4 | 7 | NW | M | 42 | - |
| 8 | DFU | M | 74 | 11.4 | 8 | NW | F | 49 | - |
| 9 | DFU | F | 81 | 10.9 | 9 | NW | F | 31 | - |
| 10 | DFU | F | 62 | 8.4 | 10 | NW | M | 76 | - |
| 11 | DFU | M | 71 | 12.5 | 11 | NW | M | 63 | - |
| 12 | DFU | M | 70 | 11 | 12 | NW | M | 68 | - |
| 13 | DFU | M | 66 | 9.9 |  |  |  |  |  |
| 14 | DFU | F | 72 | 7.8 |  |  |  |  |  |

All normal patients had no medical history of diabetes and did not suffer from general infection or cardiovascular or renal diseases. DFU, diabetic foot ulcer. NW, normal wound. M, male. F, female. HbA1c, Hemoglobin A1C.

| **Table S2 The sequences of primer pairs used in RT-qPCR.** | |
| --- | --- |
| **Gene name** | **Sequences** |
| Human PDK4 | Forward (5’-3’): AACCGTATTTCTACTCGGATGCT |
|  | Reverse (5’-3’): ACTCAAAGGCATCTTGGACCAC |
| Human β-actin | Forward (5’-3’): GTGGCCGAGGACTTTGATTG |
|  | Reverse (5’-3’): CCTGTAACAACGCATCTCATATT |
| Mouse PDK4 | Forward (5’-3’): AGTGAACACTCCTTCGGTGC |
|  | Reverse (5’-3’): TCGAACTTTGACCAGCGTGT |
| Mouse β-actin | Forward (5’-3’): GGCTGTATTCCCCTCCATCG |
|  | Reverse (5’-3’): CCAGTTGGTAACAATGCCATGT |
| Mouse P53 | Forward (5’-3’): GTGCTCACCCTGGCTAAAGT |
|  | Reverse (5’-3’): TGAGGGGAGGAGAGTACGTG |
| Mouse PAI-1 | Forward (5’-3’): AGGATCGAGGTAAACGAGAGC |
|  | Reverse (5’-3’): TTGGTTGAGGGAATCATTCAT |
| Mouse P21 | Forward (5’-3’): TTGCCAGCAGAATAAAAGGTG |
|  | Reverse (5’-3’): TTTGCTCCTGTGCGGAAC |
| Mouse P16^INK4a^ | Forward (5’-3’): AATCTCCGCGAGGAAAGC |
|  | Reverse (5’-3’): GTCTGCAGCGGACTCCAT |
| Mouse MMP3 | Forward (5’-3’): CAAAACATATTTCTTTGTAGAGGACAA |
|  | Reverse (5’-3’): TTCAGCTATTTGCTTGGGAAA |
| Mouse IL-1β | Forward (5’-3’): TGCCACCTTTTGACAGTGATG |
|  | Reverse (5’-3’): TTCTTGTGACCCTGAGCGAC |
| Mouse IL-6 | Forward (5’-3’): GCTACCAAACTGGATATAATCAGGA |
|  | Reverse (5’-3’): CCAGGTAGCTATGGTACTCCAGAA |
