# Supplementary figures and images for: PDK4 rescues high-glucose-induced senescent fibroblasts and promotes diabetic wound healing through enhancing glycolysis and regulating YAP and JNK pathway
Source: Cell Death Discov. 2023 Nov 25;9:424. doi: 10.1038/s41420-023-01725-2 (PMC10674012; doi:10.1038/s41420-023-01725-2)

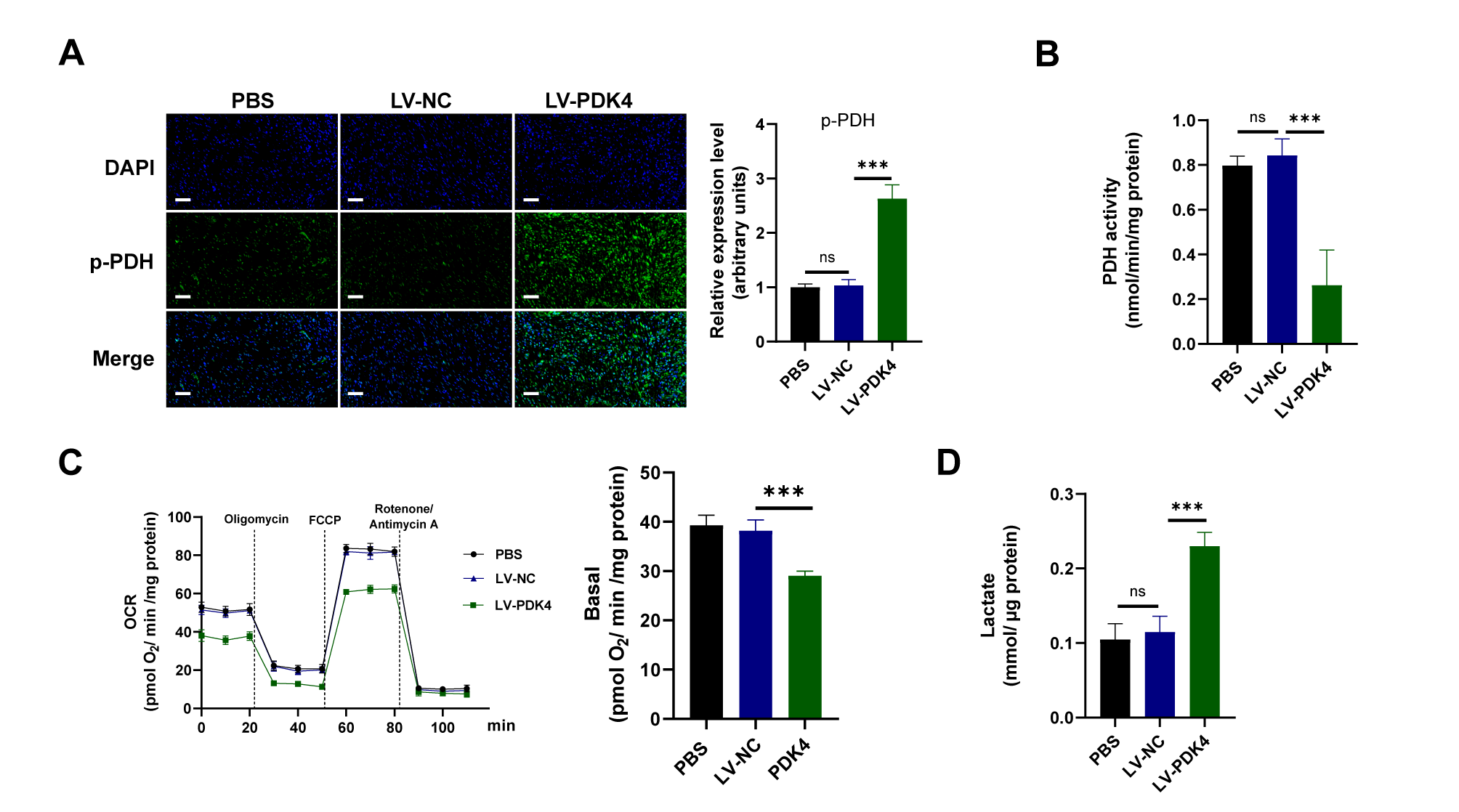

Supplement: Supplementary file 2 — Figure S1 [file 41420_2023_1725_MOESM2_ESM.tif]

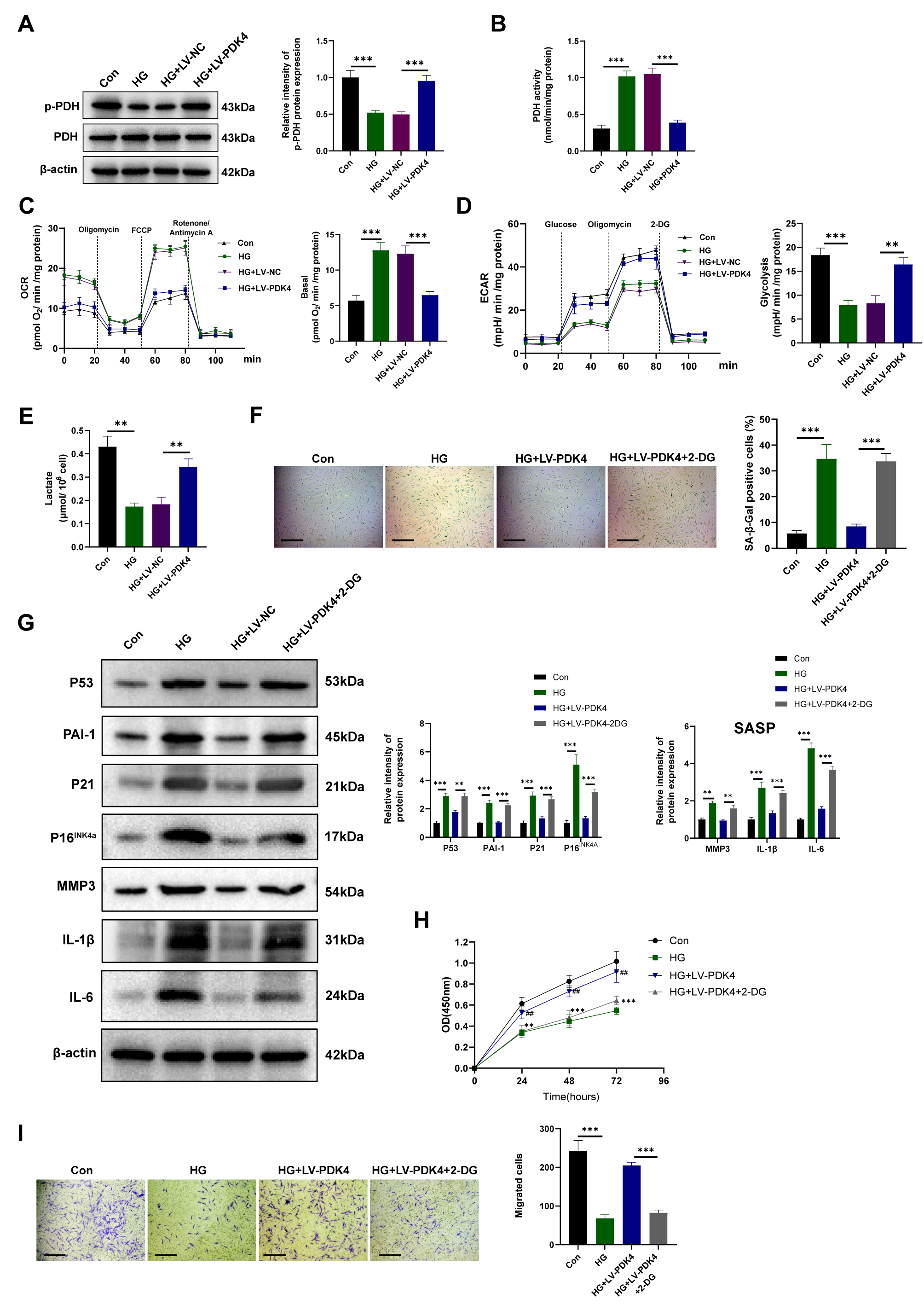

Supplement: Supplementary file 3 — Figure S2 [file 41420_2023_1725_MOESM3_ESM.tif]
